# Internal replacement of a vertebral body in pseudarthrosis—Armed kyphoplasty with bone graft-filled stents: Case report

**DOI:** 10.3389/fsurg.2023.1142679

**Published:** 2023-04-27

**Authors:** Diogo Lino Moura, Ana Rita Cavaca

**Affiliations:** ^1^ Spine Unit, Orthopedics Department, Coimbra University Hospital, Coimbra, Portugal, Coimbra, Portugal; ^2^ Anatomy Institute and Orthopedics Department, Faculty of Medicine, University of Coimbra, Coimbra, Portugal; ^3^ Orthopedics Department, Coimbra University Hospital, Coimbra, Portugal

**Keywords:** vertebral necrosis, pseudarthrosis, armed kyphoplasty, stents, bone graft

## Abstract

**Background:**

Post-traumatic vertebral necrosis and pseudarthrosis represents one of the most concerning and unpredictable challenges in spinal traumatology. The evolution of this disease at the thoracolumbar transition usually courses with progressive bone resorption and necrosis, leading to vertebral collapse, retropulsion of the posterior wall and neurological injury. As such, the therapeutic goal is the interruption of this cascade, seeking to stabilize the vertebral body and avoid the negative consequences of its collapse.

**Case description:**

We present a clinical case of a pseudarthrosis of T12 vertebral body with severe posterior wall collapse, treated with removal of intravertebral pseudarthrosis focus by transpedicular access, T12 armed kyphoplasty with VBS® stents filled with cancellous bone autograft, laminectomy and stabilization with T10-T11-L1-L2 pedicle screws. We present clinical and imaging detailed results at 2-year follow-up and discuss our option for this biological minimally invasive treatment for vertebral pseudarthrosis that mimics the general principles of atrophic pseudarthrosis therapeutic and allows to perform an internal replacement of the necrotic vertebral body, avoiding the aggression of a total corpectomy.

**Conclusions:**

This clinical case demonstrates a successful outcome of the surgical treatment of pseudarthrosis of vertebral body (mobile nonunion vertebral body) in which expandable intravertebral stents allow to perform an internal replacement of the necrotic vertebral body by creating intrasomatic cavities and filling them with bone graft, obtaining a totally bony vertebra with a metallic endoskeleton, which is biomechanically and physiologically more similar to the original one. This biological internal replacement of the necrotic vertebral body technique can be a safe and effective alternative over cementoplasty procedures or total vertebral body corpectomy and replacement for vertebral pseudarthrosis and may have several advantages over them, however long-term prospective studies are needed in order to prove the effectiveness and advantages of this surgical option in this rare and difficult pathological entity.

## Introduction

Avascular necrosis of the vertebral body diagnosis in post-traumatic context has been increasing, probably due to population aging, being more commonly found in the thoracolumbar transition and in elderlies with osteoporosis (1–7). It is estimated that posttraumatic vertebral necrosis is underdiagnosed and that its real incidence is significant, with studies indicating its occurrence in 7 to 37% of vertebral compression fractures, affecting more frequently the more comminuted fractures, those with greater flattening and the ones that reach the less vascularized regions of the vertebral body. All of these are risk factors known for the development of pseudarthrosis in general. Post-traumatic vertebral necrosis represents a failure in vertebral bone healing and, thus, it makes sense that the treatment aims to interrupt this disease's evolution and negative consequences, which represents one of the most concerning and unpredictable challenges in spinal traumatology. This way, patients with symptomatic vertebral necrosis (axial pain and functional limitation), with or without neurological compression symptoms, are candidates for surgical intervention, ranging from vertebroplasty, kyphoplasty, posterolateral arthrodesis to corporectomy and application of an intersomatic spacer. The indications for each type of surgical intervention depend on the integrity of the vertebral body, spinal stability, the patient's previous functional condition and the degree of future solicitation of the spine for each patient, which can justify only a percutaneous cementoplasty or a more invasive intervention like a total vertebral body replacement. The risks-benefits of each surgical solution must be weighed taking into account the level of functional demand of each patient and the type of vertebral necrosis, however the exact indications remain poorly defined in the literature (1, 2, 8–11). Expandable intravertebral implants are self-expanding devices applied percutaneously with posterior transpedicular access. They are introduced inside the vertebral body (armed kyphoplasty) and their expansion allows for restoration of their height, integrity and stability, when filled with bone cement or graft (12–22). The evolution of indications for these recent devices has also shown promising results in vertebral fractures evolving symptomatically and chronically to non-union situations (23, 24).

## Case presentation

**Figure 1:**
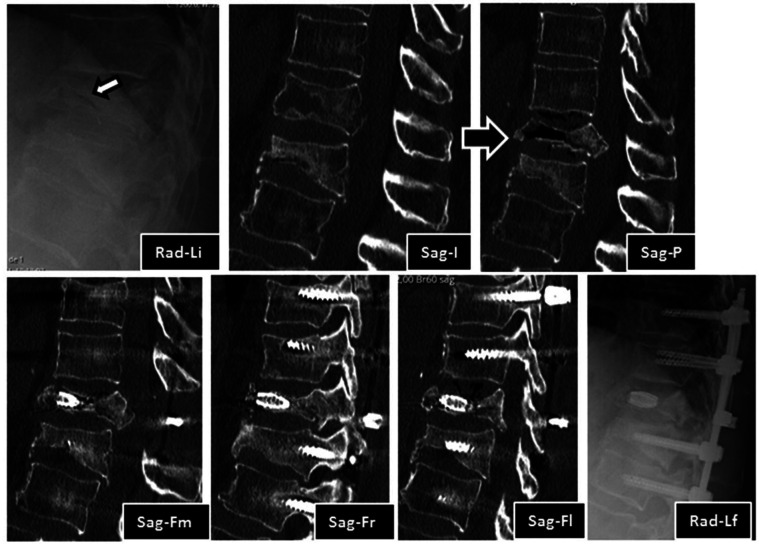
Imaging evolution of the clinical case in lateral view radiography images and sagittal CT sections: Rad-Li—initial radiograph in lateral view, showing T12 and L1 vertebral body flatenning and extensive bone destruction at T12 (arrow); Sag-I—initial median sagittal view of computed tomography, showing acute T12 fracture and old L1 fracture. Note the extensive bone trabeculae destruction at anterior half of the vertebral body; Sag-*P*—Median sagittal view of tomography, showing T12 vertebral body pseudarthrosis with a large anterior intravertebral cleft and marked posterior wall retropulsion; Sag-Fm—Median sagittal view of CT at 1 year after surgery, showing T12 vertebral body pseudarthrosis filled with stent and signs of T12 laminectomy; Sag-Fr—Right parasagittal view of CT at 1 year after surgery, showing the right stent filled with bone graft and the right pedicular screws, note the pseudarthrosis healing; Sag-Fl—Left parasagittal view of CT at 1 year after surgery, showing the left stent filled with bone graft and the right pedicular screws. Note the pseudarthrosis healing; Rad-Lf—Final radiograph in lateral view at 2 years after surgery, showing T12 stents, adjacent pedicle screws, rods and crosslink.

**Figure 2:**
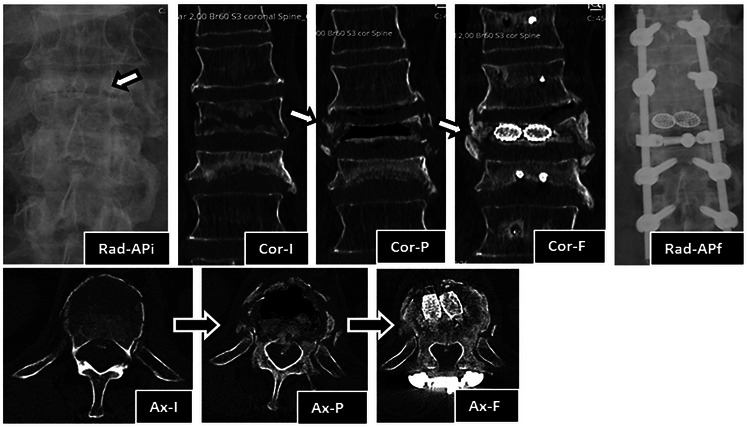
Imaging evolution of the clinical case in anteroposterior view radiography and coronal and axial CT sections: Rad-APi—initial radiograph in anteroposterior view, showing T12 and L1 vertebral body flattening and extensive bone destruction (arrow) at T12; Cor-I—initial coronal view of CT, showing acute T12 fracture and old L1 fracture. Note the extensive bone trabeculae destruction across the entire width of the vertebral body; Cor-*P*—Coronal view of CT, showing T12 vertebral body pseudarthrosis with a large intravertebral cleft across the entire width and height of the vertebral body; Cor-F—Coronal view of CT at 1 year after surgery, showing T12 vertebral body pseudarthrosis cleft filled with stents with bone graft inside, which demonstrates signs of bone healing and osteointegration. Also note the development of lateral osteophytes that help to stabilize the vertebral body to the adjacent ones; Rad-APf—Final radiograph in anteroposterior view at 2 years after surgery, showing T12 stents, adjacent pedicle screws, rods and crosslink; Ax-I—Initial axial view of CT, showing acute T12 fracture. Note the extensive bone trabeculae destruction across the entire width of the anterior half of the vertebral body; Ax-*P*—Axial view of CT, showing T12 vertebral body pseudarthrosis with a large intravertebral cleft across the entire width of the anterior half of the vertebral body; Ax-F—Axial view of CT at 1 year after surgery, showing T12 vertebral body pseudarthrosis cleft filled with two stents with bone graft inside, which demonstrates signs of bone healing and osteointegration. Also note T12 laminectomy procedure, the crosslink applied at that level and the remodeling of posterior wall retropulsion with reabsorption of intracanalar bone.

**Figure 3:**
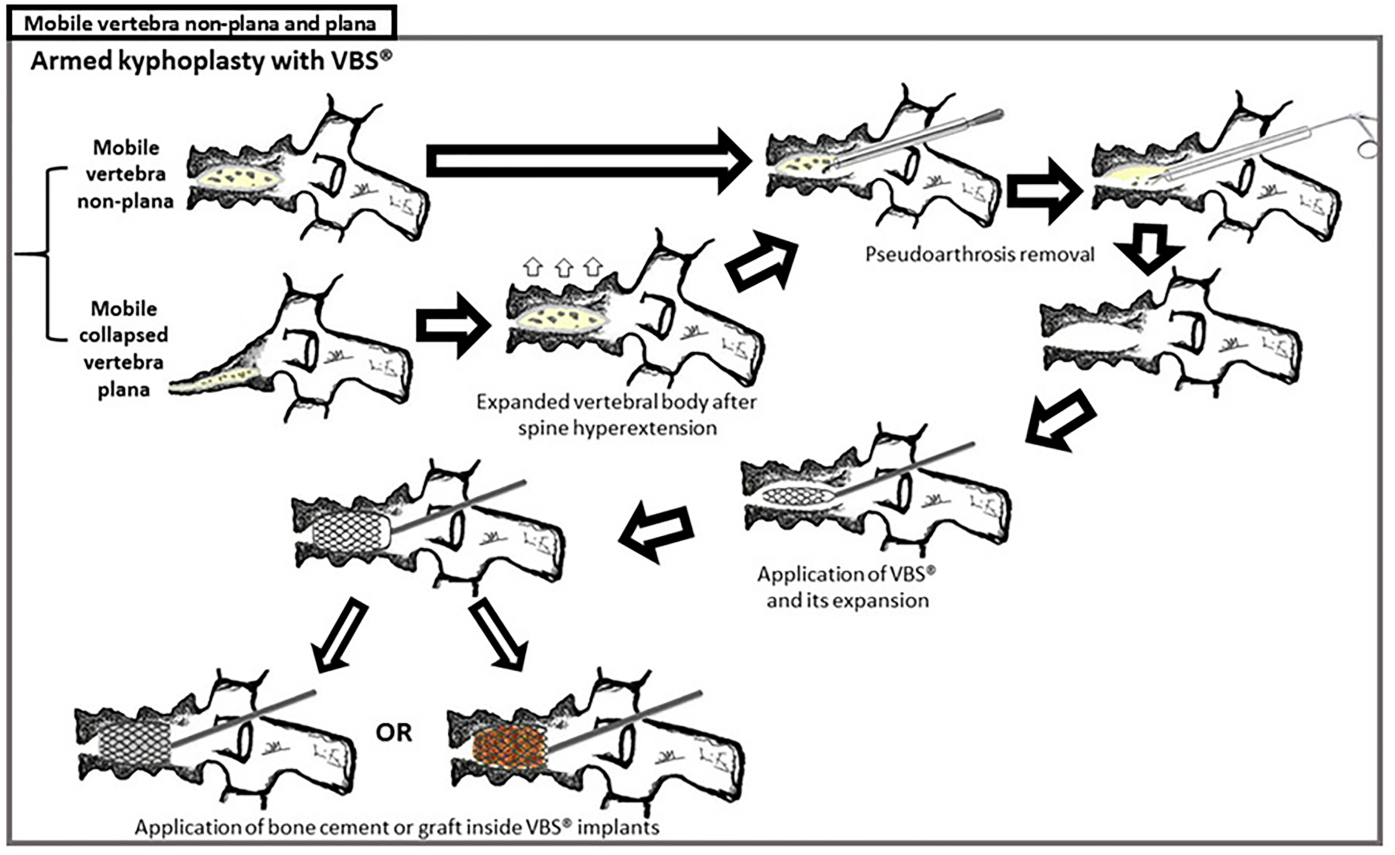
Armed kyphoplasty with VBS® in treatment of posttraumatic vertebral necrosis with mobile vertebrae non-plana and plana. The present clinical case was a mobile vertebra plana; however, the treatment of armed kyphoplasty is similar to the one of plana or non-plana mobile necrotic vertebrae, as is illustrated in this figure. After removal of pseudarthrosis region (the same as the intravertebral cleft) and proper intravertebral cleaning, the implants are expanded and then filled with bone cement or graft (in this clinical case, we chose bone graft).

We present a 71-year-old male patient, previously autonomous in daily living activities, with a history of type II diabetes mellitus, arterial hypertension and dyslipidemia, who came to our center emergency department bedridden with complaints of thoracolumbar axial pain. This pain was severe (grade 7/10 on *Visual Analog Pain Scale*—VAS) and had progressively worsened over 2 weeks, leading to the patient being currently unable to sit or walk (25). The patient had no radiculalgia or neurological deficits and the assessed *Oswestry score* (ODI) was 96% (26). The patient reported that 4 months before he had been diagnosed in another hospital with a fracture of T12 following a fall from standing height and he started conservative treatment with Jewett-type brace and analgesia. After 2 months of treatment, the pain disappeared, so the patient stopped using the Jewett-type brace and did not return to hospital. The patient brought the initial radiography and computed tomography (CT) performed at another hospital, which demonstrated an acute compression fracture of T12 vertebral body, with marked destruction of the intrasomatic trabeculae, especially in the anterior half of the vertebral body, as well as an old fracture of the L1 lower body endplate (Figure 1 Sag-Li, Sag-I and Figure 2—Rad-APi, Cor-I, Ax-I). A CT scan was performed in the context of the current episode and a pseudarthrosis of T12 vertebral body was identified, with almost total somatic collapse, the presence of a large anterior intrasomatic cleft and marked retropulsion of the posterior wall (Figure 1 Sag-P and Figure 2—Color-P, Ax-P). Average Hounsfield units at T11 and L1 and L2 vertebral body on this CT was 180, so patient demonstrated normal bone mineral density. Once this was a previously autonomous patient with current inability to verticalize the trunk due to severe axial pain in the context of T12 vertebral body pseudarthrosis and collapse, we proceeded to the following surgical intervention: laminectomy of T12 for spinal cord decompression, cleaning and removal of intravertebral pseudarthrosis focus with curettes and tweezers by bilateral transpedicular access, T12 armed kyphoplasty with VBS® stents filled with cancellous bone autograft (after the maximum expansion of the stents, we applied and impacted the bone autograft through transpedicular cannulas inside both stents until they were completely filled; autologous bone graft removed from the spinous and laminae after decompression and iliac bone) and stabilization with T10-T11-L1-L2 pedicle screws. In Figure 3 we show an illustration of the armed kyphoplasty with VBS® stents. We only performed the open median posterior lumbar approach centered on T12, strictly necessary for the T12 laminectomy and cruentation of the adjacent lamina and zygapophysis, in order to promote posterolateral arthrodesis of the T11-T12-L1 segment, while all the remaining pedicle instrumentation was performed by percutaneous approach. The patient walked on the first postoperative day and was discharged 1 week after. At the 2-month follow-up visit, he already had no relevant pain complaints and no limitations in activities of daily living, with evaluated VAS of 1 and an ODI of 12% at this time. We performed a control CT at the end of the first year after the surgery, in which we could verify the complete healing of the pseudarthrosis, with no signs of migration or failure of intrasomatic stents or pedicle screws, as well as of bone graft resorption, which indicates its osseointegration and healing (Figure 1 Sag-Fm, Sag-Fr and Sag-Fl; Figure 2 Cor-F and Ax-F). At 2-year follow-up, the patient was satisfied, pain free (VAS 0) and without relevant limitations in activities of daily living, with an assessed ODI of 4%. We present the final radiographic control at 2 years postoperatively, which demonstrates maintenance of the integrity of the vertebral body and implants (Figure 1 Rad-Lf and Figure 2 Rad-APf).

## Discussion

In the present clinical case, the marked destruction of the intrasomatic trabeculae in the initial fracture associated with its location in the thoracolumbar transition, a region of important mobility, would, in our opinion, be a criterion for performing an *ad inicium* T12 armed kyphoplasty in order to guarantee the anterior column support, stabilize it and thus precisely prevent vertebral collapse due to non-union. The repeated excessive loads on the mobile thoracolumbar transition, in view of the weakened fractured T12 vertebral body with marked destruction of the anterior column, led to insufficient stability to provide bone healing, which led to progressive bone reabsorption and necrosis, with consequent loss of its structural integrity and support function, following vertebral flattening and collapse with retropulsion of the posterior wall and neurological risk (1, 2, 8–11). During the first two months of conservative treatment, the use of Jewett brace ensured some stability to the thoracolumbar transition and, together with analgesia, attenuated the symptoms; however, the non-union and progression to pseudarthrosis led to worsening pain mainly due to intravertebral instability. Vertebral lack of stability led to progressive bone resorption and the appearance of an intrasomatic cavity or focus of pseudarthrosis, which means pathological intravertebral mobility clinically characterized by axial mechanical pain. The non-interruption of the natural course of this case of vertebral pseudarthrosis, which presents several risk factors for unfavorable evolution, such as being located at the mobile thoracolumbar transition, reaching the posterior wall and with the presence of a large intravertebral cleft, would certainly lead to progressive vertebral collapse, accentuation of posterior wall retropulsion and severe neurologic damage (1, 2, 4, 5, 8, 27, 28).

**Figure 4:**
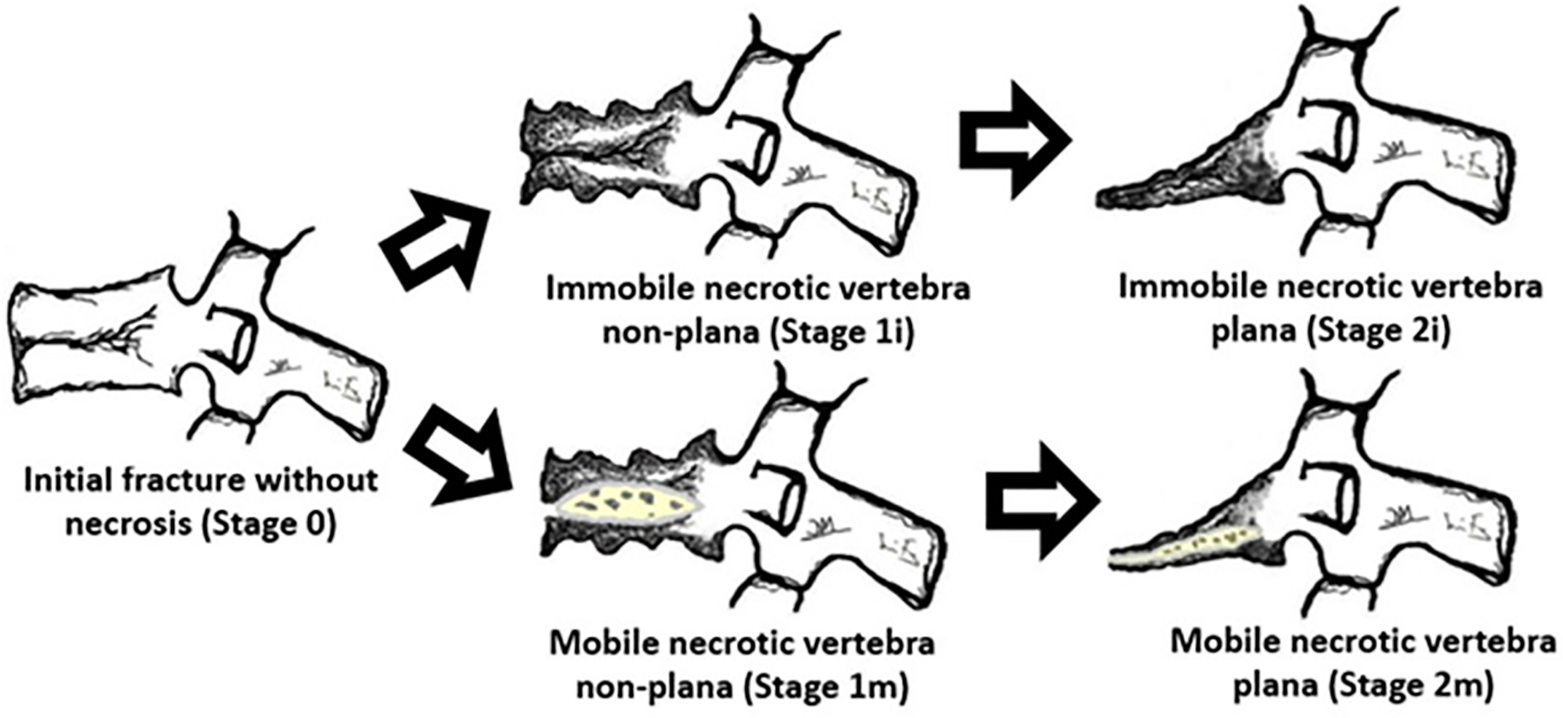
Suggested post-traumatic vertebral necrosis evolution stages: stage 0—initial fracture without necrosis; stage 1i—Immobile necrotic vertebra non-plana; stage 1 m—Mobile necrotic vertebra non-plana; stage 2i—Immobile necrotic vertebra plana; stage 2 m—Mobile necrotic vertebra plana*;* highlighting the presence of intravertebral cleft only in the mobile vertebrae marked with m(obile). Immobile vertebrae do not present intravertebral cleft and are marked with (i)mmobile. The drawings on the right side demonstrate vertebral body pseudarthrosis or mobile necrotic vertebra morphology and biomechanics. The determination of vertebral morphology and mobility in the context of post-traumatic necrosis must be performed through the combination of radiographs, including dynamic radiographs in hyperextension and orthostatism, CT and magnetic resonance imaging, also allowing to evaluate the amount of the remaining bone tissue.

Based on the scarce scientific literature available, the authors propose post-traumatic vertebral necrosis evolution stages (Figure 4) built on the grounds of parameters that directly influence the surgical therapeutic guidance based on the possibility or not to preserve the vertebral body, namely the morphology and dynamics of the necrotic vertebra (29–34). We distinguish, therefore, two types of vertebral morphology, the situations of vertebra non-plana and vertebra plana (defined as height inferior than one third of the height of the original body along its entire length), as well as two types of mobility, vertebrae with mobile deformity or pseudarthrosis (1–10, 28).

In mobile vertebrae (pseudarthrosis, that is, with intravertebral clefts—Figure 4), such as the present clinical case, regardless of their non-plana or plana morphology, it is possible to restore at least a part of vertebral body height through the positioning of the spine in hyperextension, which causes the separation of the upper and lower halves of the pseudarthrosis, increasing the size of the cleft and restoring the vertebral body height, which is filled internally (Figure 4). Thus, in these cases, a vertebral body still with sufficient bone tissue, namely with preserved bone cover (cortical ring and endplates), allows for containing the application of expandable intravertebral implants, permitting a vertebral body interior reconstruction instead of its total replacement. As such, in the face of necrosis with this vertebral dynamics, we recommend an armed kyphoplasty, in which expandable intravertebral implants will fill the empty cavity within the vertebral body surrounded by bone trabeculae impacted by the devices, and the body is then filled with bone cement or graft, which provides it with interior consistency and stability. The complete filling of the intrasomatic cleft is essential to stabilize the vertebral body, eliminating pathological and symptomatic intravertebral mobility (1–10, 28). Our clinical case was a mobile necrotic vertebra plana, which corresponds to Stage 2 m in Figure 4.

Authors usually choose VBS® stent implants in vertebrae with mobile deformity (Figure 3 and Table 1), an implant with a high capacity for space occupation, allowing the creation of large intrasomatic cavities with a cover made of the metallic mesh of the devices and impacted bone trabeculae, which allows for the application of a greater amount of bone cement or graft (12–18, 20, 21). The application of bone cement aims to fill and stabilize the interior of the vertebral body in an inert way, not expecting bone healing, solving the problem of bone regeneration inability. However, the authors defend, in post-traumatic vertebral necrosis in active patients with non-osteoporotic bone, instead of bone cement, the intrasomatic application of cancellous bone graft associated with expandable implants, seeking to obtain bone matrix colonization by osteoprogenitor cells, its vascular invasion and osseointegration, with the objective of achieving a vertebra that is biomechanically and physiologically more similar to the original in terms of loads distribution towards an active patient with a high functional demand in the future. We use autologous cancellous graft extracted regionally after laminectomy or from the patient's iliac bone for intrasomatic filling, and, if necessary, to obtain more quantity, we mix the autograft with cancellous allograft from bone bank. In the same way of the treatment concerning general bone pseudarthrosis, in vertebral necrosis we sought to use a type of bone graft combining all the properties of osteoconduction, osteoinduction, osteointegration and osteogenesis that are favorable to bone healing, which is the autologous one (27, 35–42). The application of the bone graft combined with expandable intravertebral implants not only ensures the maintenance of vertebral height in time but also protects the bone graft from excessive loads, minimizing its damage and resorption until its osseointegration is achieved, allowing to obtain a totally bony vertebra with a metallic endoskeleton. The limited histological evidence carried out in cases without the use of intravertebral implants demonstrated, in some patients, the absence of intrasomatic graft integration, with frequent microscopic findings of partial graft necrosis even in the presence of clinical and imaging signs of bone healing. This suggests a likely excessive load on the not yet osseointegrated graft (not protected by the intravertebral implant) and a weak histology-clinical correlation. Other studies have demonstrated the efficacy and revascularization of bone grafts applied in the context of vertebral pseudarthrosis (27, 42–49). The use of cancellous autograft as a method of intrasomatic filling inside the stents makes it possible to guarantee a completely bony vertebra with a metallic endoskeleton, which constitutes a more biological treatment of vertebral pseudarthrosis compared to the application of intravertebral bone cement, which, in addition to having a risk high level of extravasation in vertebral necrosis situations, cannot mimic the biology of bone healing, remaining as inert substance, biologically inactive and with excessive rigidity compared to adjacent levels, which in theory can favor fractures of adjacent vertebral bodies. Nevertheless, in spine, cementoplasty techniques (vertebroplasty and kyphoplasty) have been used to treat this disease, immediately stabilizing the vertebral body without waiting for bone healing (5–9). The option of not applying bone cement in this clinical case was based on the high risk of posterior leakage, given the morphology of the vertebra plana and the severe destruction and collapse of the posterior wall, but also because this was an active patient, with a non-osteoporotic resistant bone still with healing potential. Even in a 71-year-old patient with a severe vertebra plana stage pseudarthrosis, the combination of a proper pseudarthrosis cleaning, intrasomatic stents application and filling with bone graft allowed a sucessful internal replacement of the vertebral body, demonstrated by clear signs of bone healing and osteointegration (Figures 1, 2), which guaranteed symptoms relief. In situations of vertebral necrosis with pseudarthrosis already with marked bone resorption and vertebral collapse (vertebra plana), as in this clinical case, it is frequent, even with the positioning of the column in hyperextension and the expansion of the intravertebral implants, that only a partial height restoration is achieved and not its entirety. In this clinical case, the possible vertebral body height gain was about half of its original height; nonetheless, the stabilization and healing of the vertebral body with this morphology was enough to stop the progression of pseudarthrosis and vertebral collapse, allowing for the resolution of patient´s complaints. A proper cleaning of the pseudarthrosis region, keeping only the bone cover of the vertebral body, is essential when applying bone graft inside the stents, seeking to bring blood inside the vertebra and, as such, the necessary mediators to provide invasion by vessels of the bone graft matrix, and guarantee its desired osseointegration, without interference from interposed necrotic tissues and the fibrocartilaginous membrane that characterizes the false joint and that internally lines the intravertebral cleft, making local blood access difficult (Figure 3) (1–10, 13–18, 27, 50). In this clinical case, given the accentuated posterior wall retropulsion with compression of the medullary cord and even in the absence of neurological deficits, we initially chose to perform local prophylactic laminectomy in order to obtain the greatest possible neurological decompression. Also, this act helps to easily identify with direct visualization the pedicle entry points, which can be difficult by anteroposterior fluoroscopy because of the severe vertebral body destruction. Besides that, laminectomy allows to obtain regional bone autograft with excellent properties for intrasomatic application to seek consolidation of pseudarthrosis. The decompression performed and the extensive vertebral body bone destruction, as well as the total collapse of the posterior wall, determined the option for posterolateral arthrodesis of the T11-T12-L1 segment and percutaneous pedicle instrumentation two levels above and below, seeking to stabilize the vertebra as much as possible, reducing the loads on the posterior wall in order to minimize the risk of worsening its intracanalar retropulsion.

**Table 1:** Biomechanical characteristics of the expansive intravertebral implants VBS® (*vertebral body stenting*) (12–22).

**Implant name**	VBS® *(Vertebral Body Stenting)*
**Illustration**	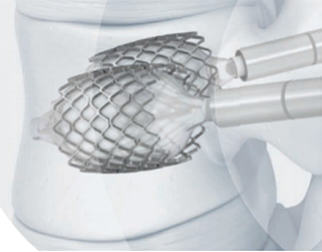
**Morphology**	Cylindrical shape network (stent). Two implants by bipedicular access
**Material**	Chromium-cobalt
**Expansion direction**	Circumferential and centrifugal in the coronal plane (craniocaudal + lateral)
**Expansion mechanism**	Hydraulic mechanism, through a kyphoplasty balloon (controlled pressure and volume)
**Expansion force**	Maximum pressure of 30 Atm; Maximum expansion volumes: #Small stent = 4 ml; #Medium stent = 4.5 ml; #Large stent = 5 ml
**Goal**	Vertebra reduction and space occupation
**Rationale**	The application of expandable intravertebral implants, also known as armed kyphoplasty, in addition to allowing the immediate analgesia and stabilization benefits of vertebroplasty and kyphoplasty, also theoretically guarantees, through a metallic endoskeleton, a greater strength of the vertebral body and a long-term maintenance of restored vertebral height. This happens because vertebral endplates, after reduction, are mechanically supported by the expanded devices, decreasing or preventing vertebral flattening after its expansion and also lowering the risk of post-traumatic local and segmental kyphosis, while ensuring very stable anterior support at the vertebral body. VBS® is a reduction and space-filling implant system since it can multidirectionally expand (vertically and laterally). It is indicated for internal replacement/reconstruction of the vertebral body, preserving its bone cover, which must be enough to contain the expansive implants and the bone cement or graft. Stents are implants that by its expansion form two big cavities within the vertebral body, coated by a casing of surrounding impacted trabeculae. These implants form cavities that, after being filled with bone cement or graft, replace much of the vertebral body interior, filling and stabilizing it. Moreover, they minimize cement extravasation by recreating the walls of the vertebral body by impaction of bony trabeculae containing the cement.

In this way, we consider this surgical technique a useful minimally invasive biological option that preserves the vertebral body in vertebral pseudarthrosis, avoiding corpectomy, which is thus only reserved for situations of non-union with immobile vertebra plana characteristics (Figure 4), that is, without pseudarthrosis, without intravertebral cleft, therefore without the possibility of increasing the vertebral height and of applying any support inside it (38, 41, 43, 50–55).

In view of this suggested post-traumatic vertebral necrosis evolution stages, it is easily understood that we should early intervene in situations of post-traumatic vertebral necrosis, ideally in vertebrae non-plana stages (stages 1i and 1 m—Figure 2), so that there is still enough bone tissue in the vertebral body to allow for the less invasive treatment, with percutaneous access and faster convalescence, which is the armed kyphoplasty. A late diagnosis or an unnecessary postponement of surgical intervention causes bone necrosis and resorption to progress, leading to situations of vertebra plana (stages 2) and increasing the risk of developing neurological damage due to retropulsion of the posterior wall and collapse of the vertebral body, which requires more aggressive surgical solutions. However, even the percutaneous current vertebral body reconstruction technique is not risk-free, and there may be migration or failure of intrasomatic stents or pedicle screws, as well as of bone graft resorption, which indicates failure to obtain osseointegration and healing of pseudarthrosis. It is also possible that the stents don't expand and as such it is not possible to put any bone inside them filling the vertebral body, which leads us to reinforce the indication of armed kyphoplasty only in the mobile vertebra plana (pseudarthrosis) and not in the rigid vertebra plana. Attempting to place expandable intravertebral implants in this type last of vertebrae involves high risks and may have serious consequences, from migration of the implants, because they are not stable within bone tissue, with damage to major neurological and vascular tissues. Also this technique requires some experience and a learning curve both in transpedicular access to the vertebral body and in percutaneous techniques with fluoroscopy.

In conclusion, this clinical case demonstrates that the treatment of pseudarthrosis of vertebral body, even in vertebra plana stage, can be carried out as an internal replacement of the necrotic vertebral body performed by posterior transpedicular access in which expandable intravertebral stents allow to create intrasomatic cavities, which are filled with bone graft, obtaining a totally bony vertebra with a metallic endoskeleton. This biological internal replacement of the necrotic vertebral body technique can be a safe and effective alternative over cementoplasty procedures or total vertebral body corpectomy and replacement for vertebral pseudarthrosis and may have several advantages over them, like allowing by a less invasive posterior technique to obtain a totally bony vertebra biomechanically and physiologically more similar to the original one and avoiding the risks of bone cement leakage. Our clinical case shows quite satisfactory clinical and radiographic results regarding this technique in a vertebra plana pseudarthrosis; however, long-term prospective studies are needed in order to prove the effectiveness and advantages of this surgical option in a rare and difficult pathological entity.

## Data Availability

The original contributions presented in the study are included in the article/Supplementary Materials, further inquiries can be directed to the corresponding author/s.
